# Physician emigration from Nigeria and the associated factors: the implications to safeguarding the Nigeria health system

**DOI:** 10.1186/s12960-022-00788-z

**Published:** 2022-12-20

**Authors:** Cosmas Kenan Onah, Benedict Ndubueze Azuogu, Casmir Ndubuisi Ochie, Christian Obasi Akpa, Kingsley Chijioke Okeke, Anthony Okoafor Okpunwa, Hassan Muhammad Bello, George Onyemaechi Ugwu

**Affiliations:** 1 Department of Community Medicine, Alex Ekwueme Federal University Teaching Hospital, Abakaliki, Ebonyi State Nigeria; 2 Department of Community Medicine, Ebonyi State University, Abakaliki, Nigeria; 3 Department of Community Medicine, University of Nigeria Teaching Hospital, Ituku-Ozalla, Enugu State Nigeria; 4 Nigeria Field Epidemiology and Laboratory Training Programme, Abuja, Nigeria; 5 Department of Obstetrics and Gynaecology, Enugu State University Teaching Hospital Parklane, Enugu, Nigeria; 6 Department of Community Medicine, Federal Teaching Hospital, Gombe, Gombe State Nigeria; 7 Department of Obstetrics and Gynaecology, University of Nigeria, Nsukka, Enugu State Nigeria; 8 Enugu State Primary Health Care Development Agency, Enugu, Nigeria

**Keywords:** Human migration, Physicians, Emigration, Health workforce, Doctors, Delivery of healthcare, Health systems, Nigeria

## Abstract

**Background:**

Adequate Human Resources for Health is indispensable to achieving Universal Health Coverage and physicians play a leading role. Nigeria with low physician–population ratio, is experiencing massive exodus of physicians. This study investigated emigration intention of physicians, the factors influencing it and discussed the implications to guide policy formulation and reforms, curtail the trend and safeguard the country’s health system.

**Methods:**

Through cross-sectional survey, 913 physicians from 37 States were interviewed with semi-structured questionnaire using Google form shared via WhatsApp and Telegram forums of Nigeria Medical Association. Data were analysed with IBM-SPSS version-25 and charts were created with Microsoft-Excel. Chi-square and multiple regression tests were done with *p*-value set at 0.05.

**Results:**

The mean age of respondents is 37.6 ± 7.9 years; majority of them are males (63.2%), married (75.5%) with postgraduate qualifications (54.1%) and working in public health facilities (85.4%). Whereas 13% and 19.3% are, respectively, satisfied with their work and willing to continue practice in Nigeria, 43.9% want to emigrate and 36.8% are undecided about future location of their practice. The commonest reasons for emigration are poor remuneration (91.3%), rising insecurity (79.8%) and inadequate diagnostic facilities (61.8%). Physicians working in public health facilities are 2.5 times less satisfied than their counterparts in non-public sector (AOR = 0.4; 95% CI = 0.3–0.8). Physicians in their thirties, forties and fifties are 3.5 (95% CI = 1.5–8.0), 5.5 (95% CI = 2.1–14.5) and 13.8 (95% CI = 3.9–49.3) times, respectively, more willing to retain practice in Nigeria than those younger and those satisfied with their work are 4.7 (AOR = 4.7, 95% CI = 2.9–7.4) times more willing to practice in Nigeria than those not satisfied.

**Conclusion:**

Majority of Nigerian physicians want to emigrate for professional practice and top among the push factors are poor remuneration, rising insecurity and inadequate diagnostic facilities. The observed trend portends danger to the country’s health system due to the foreseeable negative consequences of physician deficit to the system. We recommend upward review of physician remuneration, a root cause analysis of insecurity to determine workable preventive measures and increased funding of the health sector to improve the diagnostic infrastructure, retain physicians and save the health system from imminent collapse.

**Supplementary Information:**

The online version contains supplementary material available at 10.1186/s12960-022-00788-z.

## Background

The Sustainable Development Goals agenda gives recognition to Universal Health Coverage (UHC), which is individuals having access to the healthcare services they need at all times without suffering financial hardships [1]. Indispensable to achieving UHC is adequate human resources for health (HRH), characterized by equitable distribution of optimal skills mix and sufficient support and who enjoy decent work [1]. Low- and lower-middle-income countries (LMICs) generally experience greater shortages of HRH than upper-middle- and high-income countries (UHICs) despite having greater health needs [2, 3]. This discrepancy is largely sustained and widened by continuing international migration of health workers (IMHWs), commonly from LMICs as source to UHICs as destination. In 2015, the IMHWs was noted to have increased over the preceding decade, with 60% rise in the number of migrant doctors and nurses working in Organization for Economic Cooperation and Development countries [4].

Over the years, physician emigration from sub-Saharan Africa (SSA) and Nigeria in particular has continued to assume very disturbing trends [5, 6]. Between 2011 and 2015, Nigeria was the largest source of immigrant physicians who entered South Africa and accounted for approximately a quarter of foreign-born and foreign-trained doctors in Trinidad and Tobago [7]. According to Duvivier et al., Nigeria featured eight times among the 27 African medical schools that had more than 100 graduates in the United States of America (USA) physician workforce in 2015 and Nigerian physicians made up 45.02% of all international medical graduates (IMGs) practising in USA, who were educated in medical schools in SSA countries [8]. Also in 2015, 5.1% of IMGs in the medical council of Ireland register obtained their basic medical qualification from Nigeria [9]. From 2010 to 2016, an average of 600 General Practitioners emigrated annually from Nigeria and nearly 50% of the emigration was to Europe, followed by North America and Africa [7]. Between 2016 and 2018, over 9 000 medical doctors were reported to have left Nigeria in search of greener pastures in the United Kingdom (UK), USA and Canada [10].

The massive exodus of physicians from Nigeria has stalled the growth in physician population relative to the growth in the country’s population [11]. According to the World Health Organization (WHO) Global Health Workforce of medical doctors, the number of physicians and physician per 10 000 population in Nigeria as of 2018 were 74 543 and 3.8, respectively [12]. These numbers are over-estimations as they include all doctors licensed to practice, including retired ones and those who might have emigrated to other countries. In 2021, the Nigeria Medical Association (NMA) lamented that less than half of the over 80 000 doctors registered with the Medical and Dental Council of Nigeria (MDCN) were practising in the country, giving the country’s doctor-to-population ratio of 1 to between 4 000 and 5 000, against the WHO recommended 1 doctor to 600 people. Early in 2022, the Medical and Dental Consultants Association of Nigeria (MDCAN) bemoaned that over 100 medical consultants left from 17 Nigerian tertiary health institutions in the preceding two years. Few months after, the body conducted a survey among her members which showed that over 500 medical and dental consultants had left Nigeria for developed countries over the preceding 2 years and nine out of every 10 consultants with less than 5 years of experience plan to leave the country for greener pastures.

It has been projected that there will be continued acceleration in IMHWs with an escalating mismatch between the supply of and economic demand for health workers [13]. In 2017, a survey report revealed that 9 in 10 (88%) Nigerian doctors were seeking work opportunities abroad [14], and recently, Adebayo et al. reported that 57.4% of resident doctors in a tertiary hospital in South-West Nigeria had emigration intentions and 34.8% had made various attempts at emigrating [15]. A foreboding trend has been shown in more recent studies due to the common resolve of Nigerian medical students to practice medicine abroad after graduation in Nigeria [16, 17], suggesting that Nigeria is training physicians more for other countries than for her growing population. The fast-growing trend in physician emigration describes an acute situation on a neglected chronic problem in the Nigeria health sector.

To curb the ugly trend and prevent projected physician deficit in Nigeria [18], the need for national actions cannot be overemphasized. An indispensable step to effective national actions is a determination of factors promoting the trend and a reliable projection of the pattern of outflow from the country. This study investigated factors influencing physician emigration from Nigeria and emigration intention of physicians to guide policy formulation and reforms in order to safeguard the country’s health system.

## Methods

### Study area, population and design

The study was conducted among Nigerian physicians currently practising in the 36 states of the country and the Federal Capital Territory (FCT), Abuja, using cross-sectional analytic design. Nigeria is a multi-ethnic lower-middle income country [19], in SSA with estimated population of 216.7 million [20]. Geopolitically, it is divided into six zones including North-Central, North-East, North-West, South-East, South-South and South-West. Healthcare provision in Nigeria is a concurrent responsibility of the federal, state and local tiers of the government but private providers of healthcare, traditional medicine and complementary and alternative medicine practitioners have a visible role to play.

### Sample size estimation and sampling

Based on documented physician population in Nigeria and reported emigration intention [12, 15], a minimum sample size of 413, including 10% anticipated non response rate, was estimated using the Cochran formula for sample proportions as was reported by Singh and Masuku [21]. All physicians registered and currently practising in Nigeria were included in the study while those who had emigrated to other countries of the world at the time of data collection were excluded. Participants were selected using purposive sampling technique.

### Data collection

Quantitative data were collected online with a validated semi-structured, self-administered questionnaire, prepared with a google form, the link to which was shared on the WhatsApp, Telegram and Facebook platforms of the NMA and her affiliate bodies including the MDCAN, National Association of Resident Doctors (NARD) of Nigeria, Medical Women Association of Nigeria, National Association of Doctors in University Health Services, Medical and Dental Specialist Association in Basic Medical Sciences, Nigerian Dental Association and institutional Associations of Resident Doctors. Several WhatsApp, Telegram and Facebook platforms of the Association at the national, state, local and institutional chapters of the NMA and her affiliate bodies were used to reach out to the physicians. The researchers identified and engaged physicians in each state and health institutions who assisted in sharing the link to the data tool on the social media platforms for a window period of one month, after which acceptance of responses was stopped.

### Statistical analysis

The responses submitted by the respondents were downloaded from the internet into a Microsoft Excel Spreadsheet, cleaned out using the inclusion and exclusion criteria and then exported into IBM-SPSS Statistics version-25 for analysis. Charts were created using Microsoft Excel Spreadsheet version 2019. Descriptive statistics were generated using frequencies, means and standard deviation while inferential statistics were done with Chi-square and binary logistic regression tests with *p*-value set at 0.05 and confidence interval of 95%. A cut-off value of 0.2 was employed to select variables for inclusion into the logistic regression model after bivariate analyses.

## Results

**Fig. 1:**
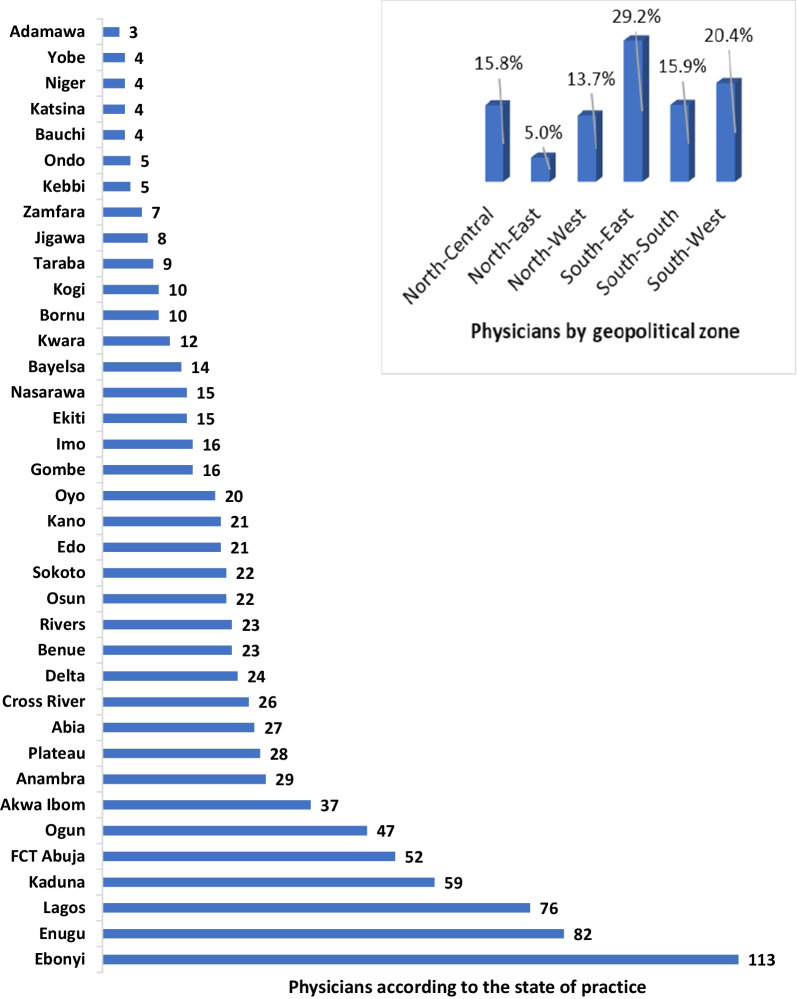
Physician’s response by geopolitical zone and state of practice in Nigeria

Of 925 responses received from 37 states of Nigeria including FCT Abuja, 913 (98.7%) met the inclusion criteria and were included in the analysis. In terms of geopolitical zones, the South-East had the highest response (29.2%) while the North-East had the least (5.0%, Fig. 1).

Table 1 shows that the mean age of the respondents is 37.6 ± 7.9 years and close to half (48.7%) of them are in the age range of 31–40 years. Majority of them are males (63.2%), married (75.5%), have postgraduate qualifications (54.1%) and obtained basic medical degree (BMD) from Nigerian (95.5%) public universities (94.7%). In terms of career progression, 21.2% of the respondents are specialist physicians or Fellows of either National Postgraduate Medical College of Nigeria (NPMCN), West African Postgraduate Medical College (WAPMC) or both, including 3.1% who have attained the status of a Professor or Reader in their areas of endeavours. Forty-point one percent of the respondents are resident doctors, including Senior Registrars (24.4%) and Junior Registrars (15.7%), who are undergoing training by the Postgraduate Medical Colleges to become specialists in their areas of interests. The mean work experience of the physicians is 10.4 with standard deviation of 7.1 years and majority of them work in public health facilities (85.3%) located mainly in urban areas (64.8%).

**Table 1:** Sociodemographic and other characteristics of the respondents

Variable	Frequency (*n* = 913)	Percent
Age in years		
21–30	203	22.2
31–40	445	48.7
41–50	215	23.5
≥ 51	50	5.5
Mean ± SD	37.6 ± 7.9^a^	
Sex		
Female	336	36.8
Male	577	63.2
Marital status		
Married	689	75.5
Never married	209	22.9
Widowed	8	0.9
Separated	7	0.8
Spouse’s profession (*n* = 689)		
Non-health worker	392	42.9
Non-physician health worker	137	15.0
Physician	160	17.5
Religion		
Christianity	734	80.4
Islam	166	18.2
Others^b^	13	1.4
Highest education		
Basic medical degree	419	45.9
Master/PhD/MD	77	8.4
Membership of a Postgraduate Medical College^c^	223	24.4
Fellowship of a Postgraduate Medical College	166	18.2
Associate Professor/Professor	28	3.1
Country of basic medical (MBBS) degree		
Nigeria	872	95.5
Abroad^d^	41	4.5
Type of institution attended to obtain basic medical degree		
Public university	865	94.7
Private university	48	5.3
Status of clinical specialization		
Medical Officers	353	38.7
Junior Registrars (Pre-Part One Trainee)	143	15.7
Senior Registrars (Post Part One Trainee)	223	24.4
Fellows (Specialist Physicians)	194	21.2
Professional work experience in years		
≤ 5	247	27.1
6–10	269	29.5
11–15	235	25.7
16–20	92	10.1
≥ 21	70	7.7
Mean ± SD	10.4 ± 7.1^a^	
Type of health institution of practice		
Public facility	779	85.3
Private facility	134	14.7
Location of practice		
Rural	73	8.0
Semi-urban	248	27.2
Urban	592	64.8

^a^Mean ± standard deviation

^b^African Traditional Religion, Eckankar, Deism, Agnostic, no affiliation

^c^National Postgraduate Medical College of Nigeria and or West African Postgraduate Medical College

^d^Ukraine (20), Russia (4), Sudan (4), Bulgaria (3), Saint Kitts & Nevis (3), India (2), Dominica (1), Oman (1), China (1), Ghana (1) and Uganda (1)

**Fig. 2:**
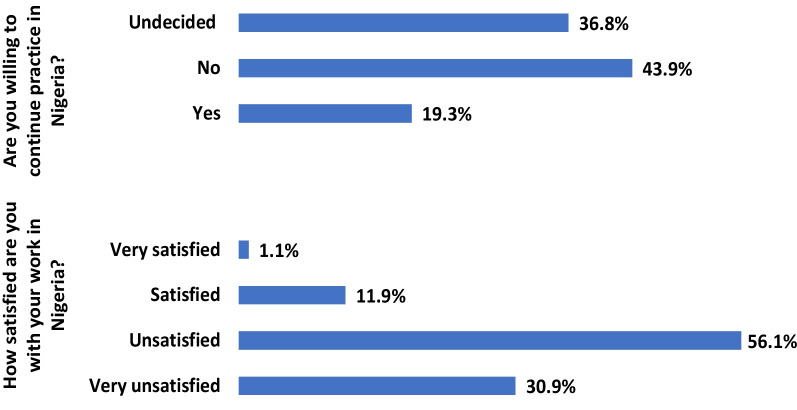
Physician’s work satisfaction and willingness to continue practice in Nigeria

**Fig. 3:**
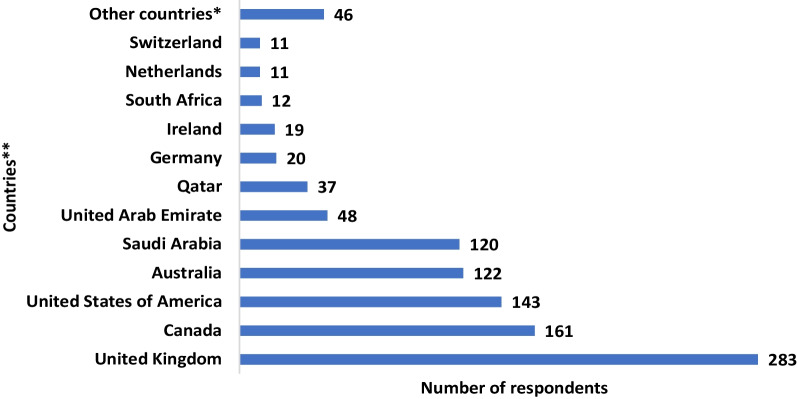
Preferred emigration destinations of physicians unwilling to continue practice in Nigeria. *Singapore, Belgium, France, Trinidad & Tobago, Grenada, Japan, Kuwait, Seychelles, China, Sweden, and Jamaica; **Multiple responses

Only 13% of the physicians are satisfied with their work and less than one-fifth (19.3%) of them are willing to continue practice in Nigeria. Out of the 80.7% who are either not willing or undecided to continue practice in the country, approximately 44 percent (43.9%) have made up their mind to emigrate to other countries of the globe for professional practice (Fig. 2). Figure 3 shows the preferred destination of the physicians with UK (283, 40.0%), Canada (161, 17.6%), USA (143, 15.7%), Australia (122, 13.4%) and Saudi Arabia (120, 13.1%) being the most preferred.

Among the few (176, 19.3%) physicians who are willing to continue practice in Nigeria, desires to maintain family ties (62.5%), contribute to the country’s health system (58.5%) and render services to the country (43.2%) and hope for a better tomorrow (42.6%) are the commonest motivating factors. Other factors are advanced age (21.8%) and a feeling that it is too late to travel abroad for professional practice (20.5%).

For the physicians with the intention to emigrate from Nigeria, poor remuneration (91.3%) is the commonest reported reason, followed by rising insecurity in the country (79.8%), having to do extra work without commensurate pay (61.8%) and lack of diagnostic equipment and facilities (61.8%). Physicians who are undecided about continuing practice in Nigeria are worried about the rising insecurity in the country (94.6%), uncertainty about government’s policies on health (92.9%), inadequacy of funding of the health sector (88.7%), inadequacy of remuneration (87.2%) and lack of diagnostic equipment and facilities (77.1%, Table 2).

**Table 2:** Factors influencing physicians’ professional practice in Nigeria

Variable	Frequency	Percent
Motivations for practice in Nigeria^a^	*n* = 176	
Desire to maintain family ties	110	62.5
Contribution to the country’s health system	103	58.5
Rendering of service to my country	76	43.2
In anticipation for a better tomorrow	75	42.6
Advanced age to travel outside for practice	38	21.6
It is considered too late to travel outside for practice	36	20.5
Good pay for service provided	1	0.6
Favourable working conditions	1	0.6

Concerns about future practice in Nigeria^a^	*n* = 336	
Rising insecurity in the country	318	94.6
Uncertainty about Government policies on health	312	92.9
Inadequacy of funding of the health sector	298	88.7
Inadequacy of remuneration for physicians	293	87.2
Lack of diagnostic equipment and facilities	259	77.1
Poor social amenities including electricity, water and road	228	67.9
Uncertainty about job security	192	57.1

Reasons for wanting to emigrate from Nigeria^a^	*n* = 401	
Poor remuneration for physicians	366	91.3
Rising insecurity in the country	320	79.8
Having to work extra hours without commensurate pay	248	61.8
Lack of diagnostic equipment and facilities	248	61.8
To gain international experience	229	57.1
To acquire further training/skills	226	56.4
Lack of insurance against work related risk	221	55.1
Lack of job security	184	45.9
Irregular pay for services rendered	166	41.4
To augment monthly income	156	38.9
Delayed pay for services rendered	146	36.4
To pursue international residency training	143	35.7
Lack of space to do specialist/residency training	99	24.7
To join family members living abroad	26	6.5

^a^Multiple responses

In Table 3, other concerns of physicians and demotivating factors for continuing practice in Nigeria are summarized into: nepotism, bad leadership and poor regulation of medical training and practice; poor relationship within the medical profession and interdisciplinary rivalries in the health sector; lack of job satisfaction with bad economic situation; and desire for improved welfare of the family including living condition, child education and health.

**Table 3:** Physicians’ self-reported concerns regarding professional practice in Nigeria

Nepotism, bad leadership and poor regulation of medical practice in Nigeria
• A profound reason is that one has to know those in political position to get a job; there is no standardized procedure on getting a job after graduation
• Lack of honesty and transparency in the Nigerian health system; jobs are given most times to those with connections while others are unemployed or employed as locum staff with the same job description and paltry pay
• Nepotism, favouritism and selfishness that has become the order of the day in most organizations in Nigeria
• Lack of political will to address the problems facing health sector in Nigeria
• There is no sincerity on the part of the leaders to make things better
• Poor regulation of medical practice by Medical and Dental Council of Nigeria (MDCN)
• Difficulty in getting placement for residency training
• Insincerity and gross failure of the government
• Uncertainty about future of both undergraduate and post graduate medical training
• Failure of implementation of regulations and extant laws guiding medical profession
Poor relationship within the medical profession and interdisciplinary rivalries in the health sector
• Hostile work environment with bad attitude of medical elders to junior doctors
• Lack of mentorship in the medical profession
• Injustice in the medical profession and the country
• Lack of job satisfactions and professional growth stagnation by those who are supposed to guide and encourage young ones in the profession
• Nigeria Medical Association’s poor welfare concern for her members
• Toxic work environment, work place bullying and harassment
• Very poor and unconducive working environment
• Lack of motivation and poor supervision from unmotivated superiors
• Antagonism of other health care workers
Lack of job satisfaction with bad economic situation
• High poverty level in Nigeria with no working system, economy, or any hope for the future
• Economic instability in Nigeria and uncertainty about the future of the country
• Lack of employment for consultants in public hospital and university
• Need for a better working system
• Dislike for current job
Desire for improved welfare of the family including living condition, child education and health
• Nigeria is becoming a failed state with no guaranteed future for my children
• Access to quality health care delivery for my family
• Better education and environment for my children
• Better life for my family
• Securing my children’s future
• Better opportunities for my children
• Family’s unity because my spouse is relocating

In Table 4, it is shown that physician’s satisfaction with professional practice is significantly associated with educational level (*χ*^2^** = **5.247, *p* = 0.002), work experience (*χ*^2^** = **13.606, *p* = 0.009), postgraduate medical college training (*χ*^2^** = **11.522, *p* = 0.009) and type of health facility of current practice (*χ*^2^** = **8.563, *p* = 0.003)*.* Physicians who work in government owned health facilities are about 2.5 times less satisfied with their practice compared to their counterparts in non-public sectors (AOR = 0.4; 95% Confidence Interval [CI] = 0.3–0.8).

**Table 4:** Factors associated with physicians’ satisfaction with professional practice in Nigeria

Variable	Satisfaction with professional practice in Nigeria (*N* = 913)		*χ*^2^ (*p*-value) on bivariate analysis	Adjusted odds ratio (95% CI) on multivariate analysis
	Satisfied *n* = 119 *n* (%)	Unsatisfied *n* = 794 *n* (%)		
Age group in years				
21–30	25 (21.0)	178 (22.4)	2.216 (0.508)	NA
31–40	53 (44.5)	392 (49.4)		
41–50	32 (26.9)	183 (23.0)		
≥ 51	9 (7.6)	41 (5.2)		
Sex				
Female	38 (31.9)	298 (37.5)	1.395 (0.238)	NA
Male	81 (68.1)	496 (62.5)		
Current marital status				
Married	96 (80.7)	593 (74.7)	2.004 (0.157)	1
Not married	23 (19.3)	201 (25.3)		0.8 (0.4–1.3)
Religion				
Christianity	91 (76.5)	643 (81.0)	2.020 (0.364)	NA
Islam	25 (21.0)	141 (17.8)		
Others	3 (2.5)	10 (1.3)		
Education				
First degree (MBBS)	43 (36.1)	376 (47.4)	5.247 (0.022*)	1
Postgraduate degree	76 (63.9)	418 (52.6)		1.7 (0.8–3.6)
Postgraduate medical training				
None	47 (39.5)	306 (38.5)	11.522 (0.009*)	1
Junior Registrars	10 (8.4)	133 (16.8)		0.7 (0.3–1.4)
Senior Registrars	25 (21.0)	198 (24.9)		0.6 (0.2–1.3)
Fellows	37 (31.1)	157 (19.8)		0.9 (0.4–1.8)
Work experience in years				
≤ 5	29 (14.4)	218 (27.5)	13.606 (0.009*)	1
6–10	23 (19.3)	246 (31.0)		0.6 (0.3–1.2)
11–15	34 (28.6)	201 (25.3)		0.9 (0.5–1.9)
16–20	20 (16.8)	72 (9.1)		1.4 (0.7–3.1)
≥ 21	13 (10.9)	57 (7.2)		1.1 (0.4–2.6)
Type of health facility of practice				
Private^a^	28 (23.5)	106 (13.4)	8.563 (0.003*)	1
Public	91 (76.5)	688 (86.6)		0.4 (0.3–0.8*)
Location of practice				
Rural/suburban	33 (27.7)	288 (36.3)	3.311 (0.069)	1
Urban	86 (72.3)	506 (63.7)		1.4 (0.9–2.2)

*CI* confidence interval, *NA* not applicable

*Statistical significance

^a^Individual, non-governmental and faith-based

Table 5 shows that age (*χ*^2^** = **99.830, *p* ≤ 0.001), marital status (*χ*^2^** = **12.566, *p* < 0.001), level of education (*χ*^2^** = **23.464, *p* < 0.001), work experience (*χ*^2^** = **108.050, *p* < 0.001) and postgraduate medical training (*χ*^2^** = **35.357, *p* < 0.001) significantly influences willingness of physicians to continue practice in Nigeria. Physicians who are in their 30s, 40s, 50s and above are 3.5 (95% CI = 1.5–8.0), 5.5 (95% CI = 2.1–14.5) and 13.8 (95% CI = 3.9–49.3) times more willing to continue their professional practice in Nigeria compared to physicians in their twenties. Junior registrars are 2 times less willing to practice in Nigeria than physicians who are not undergoing postgraduate medical training (AOR = 0.5, 95% CI = 0.2–0.9), medical doctors who have practised for 16–20 years are approximately 3 times more willing to work in Nigeria than those who have practised for 5 years or less (AOR = 2.7; 95% CI = 1.1–7.0) and those who are satisfied with their work are about 5 times more willing to continue practice in Nigeria compared to those who are not satisfied with their work (AOR = 4.7, 95% CI = 2.9–7.4).

**Table 5:** Factors associated with physicians’ willingness to continue practice in Nigeria

Variable	Willingness to continue practice in Nigeria (*N* = 913)		*χ*^2^ (*p*-value) on bivariate analysis	Adjusted odds ratio (95% CI) on multivariate analysis
	Willing *n* = 176 *n* (%)	Unwilling *n* = 737 *n* (%)		
Age group in years				
21–30	12 (6.8)	191 (25.9)	99.830 (< 0.001*)	1
31–40	66 (37.5)	379 (51.4)		3.5 (1.5–8.0*)
41–50	69 (39.2)	146 (19.8)		5.5 (2.1–14.5*)
≥ 51	29 (16.5)	21 (2.8)		13.8 (3.9–49.3*)
Sex				
Female	65 (36.9)	271 (36.8)	0.002 (0.968)	NA
Male	111 (63.1)	466 (63.2)		
Current marital status				
Married	151 (85.8)	538 (73.0)	12.566 (< 0.001*)	1
Not married	25 (14.2)	199 (27.0)		1.1 (0.6–2.0)
Religion				
Christianity	136 (77.3)	598 (81.1)	3.282 (0.194)	NA
Islam	39 (22.2)	127 (17.2)		
Others	1 (0.6)	12 (1.6)		
Education				
First (MBBS) degree	52 (29.5)	367 (49.8)	23.464 (< 0.001*)	1
Postgraduate degree	124 (70.5)	370 (50.2)		0.8 (0.4–1.6)
Postgraduate medical training				
None	59 (33.5)	294 (39.9)	35.357 (< 0.001*)	1
Junior Registrars	11 (6.3)	132 (17.9)		0.5 (0.2–0.9*)
Senior Registrars	43 (24.4)	180 (24.4)		1.1 (0.5–2.2)
Fellows	63 (35.8)	131 (17.8)		0.9 (0.4–1.8)
Work experience in years				
≤ 5	23 (13.1)	224 (30.4)	108.050 (< 0.001*)	1
6–10	28 (15.9)	241 (32.7)		0.8 (0.3–1.6)
11–15	50 (28.4)	185 (25.1)		1.4 (0.6–2.9)
16–20	39 (22.2)	53 (7.2)		2.7 (1.1–7.0*)
≥ 21	36 (20.5)	34 (4.6)		2.5 (0.8–7.6)
Type of health facility				
Private^a^	28 (15.9)	106 (14.4)	0.264 (0.607)	NA
Public	148 (84.1)	631 (85.6)		
Location of practice				
Rural/suburban	56 (31.8)	265 (36.0)	1.067 (0.302)	NA
Urban	120 (68.2)	472 (64.0)		
Satisfaction with work				
Unsatisfied	122 (69.3)	672 (91.2)	59.907 (< 0.001*)	1
Satisfied	54 (30.7)	65 (8.8)		4.7 (2.9–7.4*)

*CI* confidence interval, *NA* not applicable

^*^Statistical significance

^a^Individual, non-governmental and faith-based

## Discussion

This study investigated emigration intention of physicians and factors influencing exodus of medical doctors from Nigeria and found that majority of the doctors are not willing to continue practice in Nigeria due mainly to poor remuneration, rising insecurity and inadequate diagnostic equipment and facilities. Although the proportion of physicians who are either not willing or undecided to continue practice in Nigeria is close to 88% of Nigerian doctors previously reported to be seeking work opportunities abroad [14], the proportion of those who want to leave Nigeria is distantly below the 57.4% emigration intention reported in a study among resident doctors at the University College Hospital (UCH) Ibadan South-West Nigeria [15]. The discrepancies between our finding and those of previous studies may be due to a changing trend in emigration pattern or varying characteristics of the research participants; our respondents were drawn from several institutions in the six geopolitical zone of Nigeria and included doctors with varying age distributions, levels of training, qualifications, work experience, remunerations and possibly varying levels of job satisfaction.

Similar to a previous report [15], UK, Canada, USA, Australia and Saudi Arabia are the preferred destinations of physicians wanting to leave Nigeria. Also, another previous report of migration profile of Nigerians showed UK and the USA as the most preferred and the single most important destinations, respectively [22]. This common migration pattern is indicative of strong pull factors present in those UHICs, such as high job satisfaction among physicians in USA (91%) and UK (84%) [23], which widely varies with the relatively poor satisfaction found in our study. The high job satisfaction in those countries on the other hand may not be unconnected with the juicy remuneration for doctors in the UK [23], Canada and USA [23, 24] Australia [25, 26], and Saudi Arabia [24]. Accordingly, our finding shows that poor remuneration for doctors is a leading cause of physician emigration from Nigeria.

Good pay for physicians is a widely documented determinant of physician job satisfaction [27–30], and higher pay is known to lower physician migration [31, 32]. Compared to their counterparts in UHICs [23], Nigerian physicians are poorly remunerated. Whereas a highest-paid public sector specialist doctor on Consolidated Medical Salary Structure Level 7 Step 9 earns about N13 013 213 (US$23 660.38) per annum [33, 34], specialist physicians elsewhere earned an average of US$326 000, US$144 500 and US$128 500 per annum in USA, Germany and UK, respectively, as of 2019 [23], and between US$120 000 and US$240 000 in Saudi Arabia, United Arab Emirate and Qatar [24]. The discrepancy between these juicy pays and the paltry physician salary in Nigeria is a strong pull factor for physicians wanting to relocate their professional practice to UHICs and other countries with better pay. Worthy of note among our findings is that physicians in the public sector were two and half times less satisfied than their counterparts in the non-public sector. In a previous study, majority of physicians believed government is unconcerned with mitigating the challenges facing medical doctors in Nigeria [14]. These findings underscore the need for the government of Nigeria at all levels to put in place better incentives to retain physicians and also attract back others from the diaspora [35]. Although doctors may not have chosen medicine purely for financial reasons, earning a decent salary certainly can help to give them and their family a good quality of life.

Our findings show that worsening security situation in Nigeria is among the top reasons the doctors are emigrating. This is in concordance with the negative consequences of insecurity on health workforce motivation and availability [36, 37], which ultimately adversely affects health service delivery [38–40]. Over the years, insecurity in Nigeria has worsened [41]; in December 2021, majority (79%) of adult Nigerians opined that the country has not fared well in the area of security [42]. The worsening insecurity is attributable to many factors including Boko Haram insurgency, banditry and kidnapping, clashes between herders and farmers, clashes between Nigeria’s security forces and the Biafran separatist group, and militancy in the oil-rich Niger Delta region [41, 43]. The poor response received from the North-East region may partly be due to the paucity of doctors engendered by the protracted insecurity situation in that region [44–46]. The North-East region has been worst hit by Boko Haram insurgency with some 15 million people having been adversely affected due to their activities [45]. In 2014, more than 800 000 persons were internally displaced in the region as a result of the insurgency in Borno, Yobe and Adamawa states [22]. As of the end of 2020, the United Nations Development Programme reported that the region’s conflict due to Islamist insurgencies had killed nearly 350 000 people and projected that more than 1.1 million people may die if the conflict continues to 2030 [47]. The insecurity situation has led to destruction of more than 40% of health facilities and fleeing of many doctors from the region [48, 49].

Apart from North-East, every other region in Nigeria has had ugly experience due to worsening insecurity. The severity of insecurity is a clarion call on the federal, state and local governments to address the root causes of the problem by promoting good governance and providing jobs to the millions of educated youths and the unskilled millions of young people, thereby improving the security of the health workforce. It is also pertinent that other stakeholders in security matters such as the traditional and religious leaders sit up to their responsibility of reorienting the youth and their faithful, respectively, toward a violence-free society. This will not only help in retention of physicians and other health workforces in the country but also in attracting back more to improve the worsening physician population in the country. In a previous report, security was noted as the most important factor influencing the decision to return to Nigeria among the Nigerian health professionals living in the UK [50].

Further, the fact that inadequate diagnostic equipment and facilities demotivate Nigerian doctors shows how the dilapidated infrastructures and obsolete medical equipment in the nation’s health sector [51–53], frustrate physicians from achieving professional fulfilment. In Nigeria, modern diagnostic procedures such as Magnetic Resonance Imaging, Computed Tomography scans and other modern diagnostic procedures are still an exclusive preserve of the rich [54]. According to the 2016 National Health Facility Survey, the average availability of basic medical equipment and valid essential drugs in all health facilities country-wide were 32.9% and 34.6%, respectively, and only about one-third of surveyed health facilities received any cash grants to meet their operational costs [55]. These statistics may not have changed much recently considering the nation’s economic recessions of 2016 through 2017 and 2020 [56], and the impact of the COVID-19 pandemic.

Over the years, the paucity of skilled physicians due to brain drain, the inadequate diagnostic equipment and facilities and the underfunding of the health sector have worsened medical diagnostic and treatment challenges in Nigeria and these have in turn fueled medical tourism among the citizens [51, 52, 57], especially the political class and the rich at the expense of the poor and vulnerable populations. On the other hand, medical tourism has further reduced the revenue for sustaining the country’s local health sector due to shortage of logistics and facilities and lack of trust in medical practitioners [58]. The relationship between diagnostic and treatment challenges and medical tourism in Nigeria has assumed a positive feedback phenomenon, with one driving the occurrence of the other. If the millions of naira spent by some government officials and the Nigerian elites on medical tourism abroad are put into effective infrastructural development and Nigerians are made to receive medical treatments in the country, physicians will feel more professionally fulfilled, be better retained and huge cost of medical tourism abroad will be largely reduced. This will improve the economy and the general image of the nation’s health sector.

Our findings further show that older physicians are more willing to continue their professional practice in Nigeria compared to younger ones. This is probably due to waning interest in emigration at advanced age and more extensive family ties as the family enlarges. Older physicians may prefer to stay back at home to take care of the family and their children and rather support younger ones who may be more willing to travel abroad for greener pastures. The implication of this is that Nigeria is losing more energetic young physicians who are supposed to remain in the country to take over from the aging and retiring ones. With the current trend of physician emigration and the number of medical graduates in the country, Nigeria stands the risk of running unmanageable deficit of medical doctors in the near future. This should be a source of worry to the government of Nigeria and all stakeholders in the country’s health care industry.

In view of the already poor Healthcare Access Quality performance [59], and poor key health indicators such as high maternal mortality ratio and under-five mortality [60], emigration of physicians can literally create more life-endangering situation for Nigerians when the health services vanish due to lack of qualified personnel [61]. When there is shortage of physician to provide healthcare services to the people, a number of effects inevitably become evident in the health system, including: unnecessarily prolonged waiting time before consultations, increased workload for practitioners, time constraints on doctor–patient interactions, overworked and stressed practitioners, higher prices for consultations and lower quality of care, ultimately resulting in worsening of the health system.

Beyond weakened health system, physician emigration has negative economic consequences on Nigeria due to loss of return on investment [62, 63]. The country suffers financial losses when domestically trained physicians emigrate because the training of physicians in Nigeria is largely publicly financed. Undergraduate medical education in Nigeria is subsidized with government funds at the public universities which constitute 35 (79.5%) of the 44 accredited medical schools operational in the country [64]. The situation is the same at the postgraduate level since residency training is run mainly at the federal and state university teaching hospitals, funded with government money. According to Saluja et al., LMICs lose US$15.86 billion annually due to excess mortality associated with physician migration to UHICs and greatest total costs are incurred by India, Nigeria, Pakistan and South Africa and by the WHO African region [62]. Over a decade ago, Kirigia et al. had estimated that about US$1 854 677 is lost for every doctor that emigrates from a country in the WHO African region [63]. The loss in Nigeria is huge because majority of the physicians obtain their BMD from public universities, an investment that is now benefiting other countries.

To cushion the effects of massive exodus of doctors from Nigeria, there is need to expand medical education to produce more graduates and more pressing need to retain them by addressing the push factors for emigration. Expanding medical education requires a more robust funding of medical schools to enable more efficient admission process, procurement of medical infrastructure for learning and improvement of remuneration for clinical teachers in the medical education system [65]. This will improve the limited numbers and capacity of medical schools in the country which currently stands at 37 fully accredited and 7 partially accredited ones with total carrying capacity of 3 990 per year [64]. To retain physicians from leaving the country, there should be more funding of the health sector to improve on physician remuneration and provide adequate diagnostic equipment and facilities. With allocation of an average of 4.7% of her annual budgets to the heath sector [66], two decades following the Abuja declaration of the African Union countries to be allocating at least 15% of their annual budgets to improve the sector [67, 68], Nigerian health sector has been grossly under-budgeted.

### Limitations to the study

The authors are aware that inability to operate NMA and affiliated body WhatsApp, Telegram or Facebook platforms due to poor or failed network connectivity during data collection period may have deprived some physicians the opportunity to participate in this study and so made this report not completely representative of the true situation of things, since access to the data tool depended on good internet service. However, these challenges were mitigated by allowing a long window period of one month for submission of responses. Further, the link to the google form was regularly shared on all the identified social media platforms of NMA on two-daily basis throughout the data collection period. WhatsApp and Telegram are not only the common social media platforms of communication of NMA and her affiliate bodies, they are the official media for dissemination of information of the Association and most Nigerian physicians use at least one or two of the platforms. Another limitation is that the link to the data tool was accessible to physicians who have emigrated from Nigeria but remained in the social media platforms of NMA. This challenge was addressed by excluding the responses submitted by physicians in this category during data cleaning before analysis was conducted.

## Conclusions

Our study demonstrated that majority of Nigerian physicians are either not willing or undecided to continue professional practice in the country and besides the major push factors like poor remuneration, rising insecurity and inadequate diagnostic facilities and equipment, the physicians are concerned about poor funding of the health sector. The growing trend in physician emigration portends danger to the Nigeria health system due to the foreseeable negative consequences of physician deficit to the system. We recommend upward review of physician remuneration at the three tiers of government, a root cause analysis of insecurity to determine workable preventive measures and increased funding of the health sector to improve the diagnostic infrastructure, retain physicians and save the health system from imminent collapse.

## Supplementary Information

**Additional file 1.** Physician emigration from Nigeria survey data set.

## Data Availability

All data generated or analysed during this study are included in this published article as Additional file 1.

## Abbreviations

AEFUTHA: Alex Ekwueme Federal University Teaching Hospital Abakaliki
AOR: Adjusted odds ratio
BMD: Basic medical degree
CI: Confidence interval
FCT: Federal Capital Territory
HRH: Human Resources for Health
IMGs: International medical graduates
IMHWs: International migration of health workers
LMICs: Low- and middle-income countries
MDCAN: Medical and Dental Consultants Association of Nigeria
MDCN: Medical and Dental Council of Nigeria
NARD: National Association of Resident Doctors
NMA: Nigeria Medical Association
NPMCN: National Postgraduate Medical College of Nigeria
SSA: Sub-Saharan Africa
UCH: University College Hospital
UHC: Universal Health Coverage
UHICs: Upper and high-income countries
UK: United Kingdom
USA: United States of America
WAPMC: West African Postgraduate Medical College
WHO: World Health Organization
